# Non-invasive assessment of fatigue and recovery of inspiratory rib cage muscles during endurance test in healthy individuals

**DOI:** 10.1371/journal.pone.0277131

**Published:** 2022-12-07

**Authors:** Thiago Bezerra Wanderley e Lima, Antonio Sarmento, Rayane Grayce da Silva Vieira, Esmívany Lhara de Freitas Castro, Francesca Pennati, Andrea Aliverti, Vanessa Regiane Resqueti, Guilherme Augusto de Freitas Fregonezi

**Affiliations:** 1 PneumoCardioVascular Lab/Hospital Universitário Onofre Lopes (HUOL), Empresa Brasileira de Serviços Hospitalares (EBSERH), Universidade Federal do Rio Grande do Norte, Natal, Rio Grande do Norte, Brazil; 2 Departamento de Fisioterapia, Laboratório de Inovação Tecnológica em Reabilitação, Universidade Federal do Rio Grande do Norte, Natal, Rio Grande do Norte, Brazil; 3 Dipartimento di Elettronica, Informazione e Bioingegneria, Politecnico di Milano, Milan, Italy; Universita degli Studi di Milano, ITALY

## Abstract

**Introduction:**

Fatigue is defined as loss of capacity to develop muscle force and/or velocity that is reversible at rest. We assessed non-invasively the fatigue and recovery of inspiratory rib cage muscles during two respiratory endurance tests in healthy individuals.

**Methods:**

The sniff nasal inspiratory pressure (SNIP) was assessed before and after two respiratory endurance tests: normocapnic hyperpnea (NH) and inspiratory pressure threshold loading (IPTL). Contractile (maximum rate of pressure development and time to peak pressure) and relaxation parameters (maximum relaxation rate [MRR], time constant of pressure decay [τ], and half relaxation time) obtained from sniff curves and shortening velocity and mechanical power estimated using optoelectronic plethysmography were analyzed during SNIP maneuvers. Respiratory muscle activity (electromyography) and tissue oxygenation (near-infrared spectroscopy—NIRS) were obtained during endurance tests and SNIP maneuvers. Fatigue development of inspiratory rib cage muscles was assessed according to the slope of decay of median frequency.

**Results:**

Peak pressure during SNIP decreased after both protocols (p <0.05). MRR, shortening velocity, and mechanical power decreased (p <0.05), whereas τ increased after IPTL (p <0.05). The median frequency of inspiratory rib cage muscles (i.e., sum of sternocleidomastoid, scalene, and parasternal) decreased linearly during IPTL and exponentially during NH, mainly due to the sternocleidomastoid.

**Conclusion:**

Fatigue development behaved differently between protocols and relaxation properties (MRR and τ), shortening velocity, and mechanical power changed only in the IPTL.

## Introduction

Normocapnic hyperpnea (NH) and inspiratory pressure threshold loading (IPTL) are respiratory endurance tests (RET) used to assess respiratory muscle function. NH is performed under low resistance and high flow conditions and involves expiratory and inspiratory muscles, whereas IPTL is performed in high resistance and low flow conditions and involves inspiratory muscles [1]. These two methods may interfere differently with fatigue resistance, leading to distinct physiological responses [2].

Fatigue is characterized by decreased muscle capacity to generate strength and/or velocity during activity [3–5] and may limit exercise tolerance in healthy and diseased individuals [6, 7]. Fatigue is also influenced by the recruitment pattern, which depends on the activity performed. Recently, Sarmento et al. [8] analyzed fatigue development (70% of maximal inspiratory pressure) and recovery of sternocleidomastoid, scalene, and parasternal muscles of healthy individuals during one type of RET and observed changes in mechanical power and shortening velocity. However, literature lacks data regarding fatigue behavior between different RET modalities.

Although studies have used invasive methods to assess respiratory muscle fatigue [1, 3, 4] less painful and more accessible options are desirable in clinical practice for assessing the development of inspiratory muscle fatigue (e.g., muscle contraction and relaxation parameters, surface electromyography [sEMG], chest wall volumes, and tissue oxygenation). Contraction and relaxation parameters of inspiratory rib cage muscles may change during fatigue development [5, 9–13] and these variables can be analyzed non-invasively using sniff nasal inspiratory pressure (SNIP) [14–17]. Also, muscle fatigue can be assessed through changes in EMG frequency spectrum [1, 3, 7]. For example, the median frequency of power spectrum (MF) decreases during the development of muscle fatigue [18–20]. Moreover, the assessment of chest wall volumes is essential to understand the physiology of chest wall muscles before and after fatigue [21–23]; this can also be assessed non-invasively using optoelectronic plethysmography (OEP) [24, 25].

In recent years, tissue oxygenation and blood flow have been studied indirectly using near-infrared spectroscopy (NIRS) technique. NIRS is non-invasive and measures tissue concentrations of oxygenated hemoglobin (O_2_Hb) and deoxygenated hemoglobin (HHb); the sum of concentrations (O_2_Hb + HHb) represents total hemoglobin concentration (tHb) and is used as a substitute measure for local blood volume [26, 27]. Analyses of these variables provide indirect information on tissue oxygenation and inspiratory muscle metaboreflex, which may be involved in respiratory muscle fatigue.

Although the SNIP maneuver was used in previous studies to assess fatigue and recovery of inspiratory muscles during inspiratory resistive load [8, 11, 16], the behavior of respiratory muscles during endurance tests with different characteristics (e.g., strength and endurance) is still unknown. In this sense, we hypothesized that fatigue development of inspiratory rib cage muscles differs according to RET protocol and causes changes in contraction and relaxation parameters, shortening velocity, and mechanical power of these muscles. Therefore, we aimed to assess fatigue and recovery of inspiratory rib cage muscles using non-invasive techniques during different endurance tests in healthy individuals.

## Material and methods

### Study design and participants

This cross-sectional study included 22 healthy volunteers (10 men and 12 women; age: 18 to 29 years; body mass index: 18 to 25 kg/m^2^; and no history of smoking or respiratory, cardiac, or neuromuscular diseases). Exclusion criteria were individuals with forced vital capacity (FVC) and forced expiratory volume in the first second (FEV_1_) < 80% and FEV_1_/FVC ratio < 85% of predicted, highly active (International Physical Activity Questionnaire short version-IPAQ) [28], and with nasal congestion or deviated septum. The study followed the Declaration of Helsinki and was approved by the research ethics committee of Hospital Universitário Onofre Lopes (HUOL-EBSERH/BRASIL) according to protocol number 3.084.956.

### Respiratory endurance tests

Two different respiratory muscle training devices were used to perform NH (SpiroTiger, Idiag®, Fehraltorf, Switzerland) and IPTL (POWERbreathe, HaB International Ltd, Southam, UK). For the former, parameters were based on a previous study [29]: size of rebreathing bag, established at 50% of individual’s vital capacity; minute volume (VE), adjusted to 70% of 15-second MVV; and respiratory rate (RR), defined according to manufacturer’s recommendations (respiratory rate = AMV/[Bag size × 1.2] [1/min], where AMV is target ventilation per minute). MVV level was set at 70%, according to previous studies [2]. Individuals were asked to breathe using a pre-determined VE, and task failure was defined when they reached volitional exhaustion or were unable to maintain RR and VE after three warnings from evaluator.

IPTL was performed using a medium-resistance POWERbreathe^®^ Classic (POWERbreathe; HaB International Ltd., Southam, UK) with inspiratory load of 80% of MIP [30] (expiration was unimpeded). Individuals selected the RR and tidal volume to overcome the load imposed [31]. Task failure was defined when individuals reached volitional exhaustion or were unable to overcome the load after three warnings from the evaluator.

### Study protocol

All individuals were previously informed about the study methods, and ten SNIP maneuvers were performed before initiating the protocol to avoid learning effect. Data were collected on two days, separated by seven days; the order of RETs was randomized using simple draw with opaque envelopes.

Anthropometric data, physical activity level, pulmonary function, and respiratory muscle strength were assessed on the first day. After 20 minutes of rest, the first RET protocol was initiated with the selected device. On the second day, individuals performed RET only using the second device. Heart rate (HR) and peripheral arterial saturation (SpO_2_) were monitored during tests, whereas perceived effort (Borg scale) was assessed before and immediately after RET.

The experimental protocol consisted of three phases:

**Pre-RET phase:** individuals were asked to remain seated in a chair without back support, while a single researcher positioned the sEMG electrodes and retro-reflective markers (OEP). Subsequently, the manometer plug was inserted in one nostril (contralateral nostril remained unobstructed), and individuals were asked to perform ten SNIP maneuvers with 30-second intervals. Individuals were monitored simultaneously in this phase using OEP and sEMG. For each individual, the SNIP maneuver generating the highest peak pressure was used to analyze sEMG, OEP, and parameters obtained from sniff curve (i.e., pre-RET values).

**RET:** after pre-RET phase, individuals remained seated at rest for 15 minutes while information regarding the protocol was provided. RET was performed using the device selected for that day and sEMG and NIRS were acquired simultaneously during RET. Test duration (Tlim) was recorded at time of task failure according to previously established criteria. Verbal encouragement was provided throughout the protocol.

**Fig 1:**
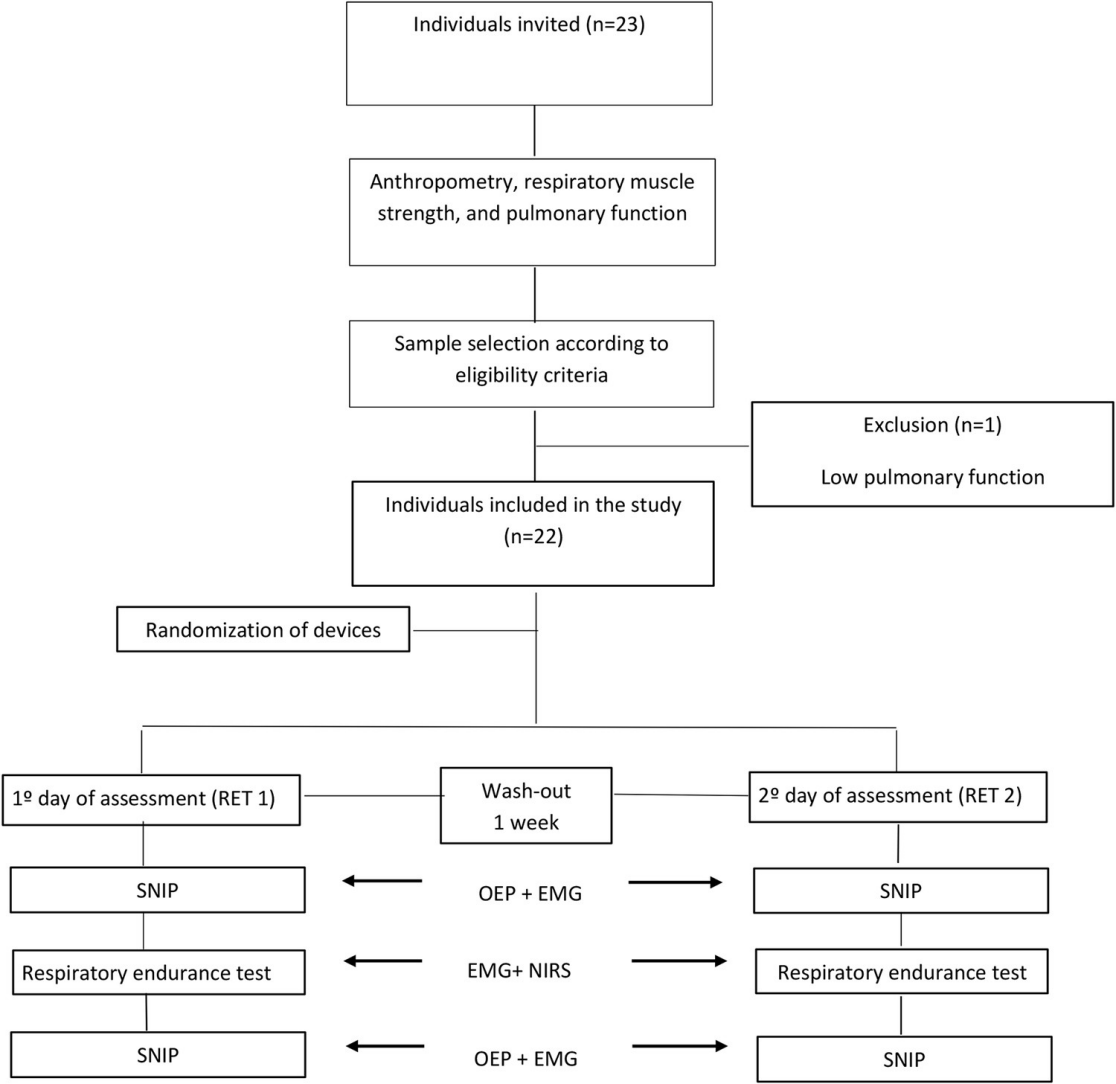
Flowchart of participant selection.

**Recovery phase (post-RET):** after test, individuals were instructed to immediately take off the device, place the manometer plug in the same nostril used previously, and perform ten SNIP maneuvers with 30-second intervals. sEMG signal was also captured simultaneously with OEP. This phase considered for analysis all values obtained in the ten maneuvers (Fig 1).

### Pulmonary function

Pulmonary function was assessed using a KoKo DigiDoser spirometer (Spire Health, Inc.; Longmont, CO, USA). FEV_1_, FVC, FEV_1_/FVC ratio, and forced expiratory flow between 25–75% of FVC maneuver were assessed. Technical procedures followed the American Thoracic Society/European Respiratory Society [32], and predicted values were calculated using reference values for the Brazilian population [33]. Slow vital capacity maneuver and voluntary maximum ventilation test for 15 seconds (MVV) were also performed.

### Respiratory muscle strength

The assessment of respiratory muscle strength was performed using a digital manometer (NEPEBLabCare/UFMG, Belo Horizonte, MG, Brazil) with individuals seated and with feet on the floor. Maximum inspiratory (MIP) and expiratory (MEP) pressures were obtained from residual volume and total lung capacity, respectively. To obtain predicted values, we used regression equations for the healthy Brazilian population [34]. Nasal expiratory pressure (SNEP) and SNIP were also measured. For SNIP test, equations were used to obtain reference values [35].

### Sniff curve analysis

All sniffs were performed at functional residual capacity with individuals seated on a chair without back support. Contraction and relaxation properties were assessed from sniff traces using custom software developed in MATLAB software (The MathWorks Inc., Natick, MA, USA). For contraction parameters, contraction time (expressed in ms) was calculated as the time to reach peak pressure [12]. Maximum rate of pressure development (MRPD) was calculated as the negative peak of the first derivative of pressure-time curve (MRPD normalized to sniff peak pressure [MRPD/sniff peak], expressed in ms^-1^) [36], and time to peak shortening (TPS) (expressed in ms) was calculated as the time to reach MRPD [37].

For relaxation parameters, the half relaxation time (½RT) (expressed in ms) was calculated as the time to reach half of the relaxation curve (i.e., between peak pressure and end of pressure generation). Maximum relaxation rate (MRR) (expressed in ms^-1^) was defined as the positive peak of the first derivative of pressure-time curve (normalized for the peak sniff pressure) [16]. The time constant (τ, tau) of the decay exponential behavior (P = exp^-t/τ^) of the lower 50%-70% pressure decay was also calculated. The correlation coefficient of the regression line of the natural logarithm of the pressure and the time should be greater than 0.98 for τ to be accepted [15]. The following criteria were used for selecting the appropriate sniffs for analysis: (1) sniff performed from functional residual capacity, (2) peak pressure maintained for less than 50 ms, (3) duration of inspiratory effort < 500 ms, and (4) SNIP waveform with smooth decay curve [13].

### Chest wall and compartmental volumes

Chest wall volumes were assessed using the OEP system (BTS Bioengineering, Quincy, MA, USA), which included eight photosensitive cameras that captured the movement variation of 89 retro-reflective markers placed over predefined regions of thorax and abdomen [38]. The device was calibrated before each measurement using a frequency of 60 frames per second to recognize markers.

Data considered for OEP analysis included inspiratory time (Ti) and changes in chest wall (ΔV_CW_), pulmonary rib cage (ΔV_RCp_), abdominal rib cage (ΔV_RCa_), and abdominal (ΔV_AB_) volume. Shortening velocity index of inspiratory rib cage (ΔV_RCp_/Ti), diaphragm (ΔV_AB_/Ti), and global inspiratory muscles (ΔV_cw_/Ti) were also calculated. In addition, the product of pressure generated during SNIP maneuver and shortening velocities (ΔV_CW_/Ti, ΔV_RCp_/Ti, and ΔV_AB_/Ti) represented mechanical power of global inspiratory muscles (Ẇ_insp_), inspiratory rib cage muscles (Ẇ_rcm_), and diaphragm (Ẇ_di_), respectively [38]. All these data were analyzed during SNIP maneuvers in the pre-RET and recovery (post- RET) phases.

### Surface electromyography

sEMG of respiratory muscles was obtained simultaneously with the assessment of chest wall volumes. An electromyograph (TeleMyo DTS Desk Receiver®; Noraxon USA Inc., Scottsdale, AZ, USA) was used to acquire signals with 16-bit resolution, at sampling frequency of 1500 Hz, with low-pass filter of 500 Hz, gain of 1000×, and common-mode rejection index of > 100 dB. Data were stored in the MR software, version 3.8 (Noraxon USA Inc., Scottsdale, AZ, USA). Ag/AgCl bipolar surface electrodes, with inter-electrode distance of 10 mm, were attached to the skin after preparation with abrasion, shaving, and cleaning (70% alcohol) [39]. This procedure was performed on the following muscles in the right side according to the direction of muscle fibers: 1) sternocleidomastoid (SCM), at the lower third of the distance between the mastoid process and sternoclavicular joint [40]; 2) scalene (ESC), at 5 cm from the sternoclavicular joint and 2 cm above this point [41]; and 3) parasternal (PS), at the second intercostal space and 3 cm from the sternum [42].

### sEMG processing and analysis

sEMG signals were processed using a 20–400 Hz 2^nd^ order Butterworth bandpass filter and analyzed in time and frequency domains to calculate the root mean square (RMS) and MF, respectively. Each portion of the sEMG signal corresponding to a SNIP maneuver in pre-RET and post-RET phases was subjected to RMS and MF analysis. Only MF was obtained during RETs, which was calculated by applying continuous wavelet transform technique using Daubechies4 mother analysis in 5-second windows. For analysis, MF was normalized for each individual by expressing it relative to values obtained at the beginning of the fatigue protocol (i.e., mean of initial 10 s) and plotted as a function of RET total time. For RMS analysis during SNIP maneuvers, the pre-RET value was designated as 100%, and all post-RET values were calculated as the percentage of this value. All sEMG analyses were performed offline using MATLAB software (The MathWorks Inc., Natick, MA, USA).

### Tissue oxygenation

Tissue oxygenation was assessed using NIRS device (Portamon; Artinis Medical Systems BV, Elst, Netherlands). This technique is based on applying a light with near-infrared wavelength, considering the principles of absorption and dispersion according to spatially resolved spectroscopy [26]. The Portamon is a non-invasive, portable, and wireless tool containing a receiver and three light-emitting diodes spaced at 30, 35, and 40 mm; we used a 40 mm distance for analysis because of greatest penetration depth, according to manufacturer. Changes in O_2_Hb, HHb, and tHb were assessed through changes in light absorption at different wavelengths (760 and 850 nm) with frequency of 10 Hz to estimate oxygenation (O_2_Hb and HHb) and local blood volume (tHb). The equipment was fixed on the skin over the left SCM (middle-distance between mastoid process and medial end of the clavicle) using adhesive tapes after cleaning with 70% alcohol [43].

### Statistical analysis

Data normality was verified using Shapiro-Wilk test. For descriptive analyses, mean and standard deviation were used for normal distribution and median and interquartile range for non-normal distribution. Comparisons between moments (pre-fatigue and recovery) and RETs were performed using two-way repeated measures ANOVA. The pre-fatigue and recovery moments of each RET (intragroup) were compared separately using Friedman’s test or repeated measures one-way ANOVA. To avoid type I error due to the multiplicity of post-fatigue moments, the two-stage false discovery rate (threshold value of 5%) was applied in case of statistical significance instead of Bonferroni or Dunn’s post-hoc test [44]. Intergroup comparisons between pre-fatigue and recovery moments were performed using the unpaired t-test or Mann-Whitney test.

Regression analysis was applied to MF to verify whether inspiratory rib cage muscles were developing fatigue during protocols, while regression curves adjusted to maximum values in a least-square sense were used as index of fatigue development. Both analyzes were conducted separately for each muscle and considering the set of inspiratory rib cage muscles assessed (SCM, ESC and PS). For all regression analyses, coefficients of determination (r^2^), slopes, and time constants were calculated during task failure (TF, from beginning to end of the RET protocol) and recovery moments (TRec, from the last point of the RET protocol to the tenth SNIP maneuver). For linear regressions, TF and TRec were calculated as the inverse values of regression slopes. For non-linear regressions, slopes were calculated as derivatives of the exponential equation at the beginning of the task failure protocol [8]. Muscle fatigue was identified if the following two criteria were met: 1) negative slope (linear regressions) [45] and 2) decrease below 60% of values recorded at the beginning of task failure [46] (exponential regressions).

NIRS variables were monitored in real-time and subsequently analyzed using the Oxysoft software (Artinis Medical Systems BV, Elst, Netherlands). A moving Gaussian filter was applied, and linear regression analyses were performed at intervals of 10% of total RET.

## Results

Twenty-two individuals (12 men and 10 women) participated in the study. Mean age was 24.36 ± 2.06 years, and mean body mass index was 22.40 ± 2.02 kg/m^2^. Table 1 presents anthropometric and pulmonary function data.

**Table 1:** Anthropometric data, absolute and predicted values of lung function, respiratory muscle strength, and physical activity level.

Individuals _(n)_	22
Age _(years)_	24.36 ± 2.06
Height_(m)_	1.71 ± 0.08
Weight _(kg)_	65.15 ± 9.61
BMI _(kg/m_^2^_)_	22.40 ± 2.02
FVC _(L)_	4.43 ± 0.82
FVC _(%pred)_	95.92 ± 8.96
FEV_1 (L)_	3.75 ± 0.65
FEV_1 (%pred)_	94.54 ± 7.45
FVC/FEV_1_	0.84 ± 0.047
FVC/FEV_1 (%pred)_	96.65 ± 7.41
FEF _25–75%(L/s)_	3.94 ± 0.7
FEF _25–75%(L/s) (%pred)_	84.7 ± 13.4
MIP _(cmH2O)_	106.3 ± 18.8
MIP _(%pred)_	90.0 ± 15.3
MEP _(cmH2O)_	113.3 ± 30.8
MEP _(%pred)_	90.6 ± 17.8
SNIP _(cmH2O)_	100.1 ± 20.2
SNIP _(%pred)_	88.0 ± 15.8
SNEP _(cmH2O)_	104.5 ± 31.4
Very active	-
Active	5 [22.7%]
Irreg. Active A	10 [45,45%]
Irreg. Active B	7 [31.8%]
Sedentary	-

Data presented as mean ± SD. FVC: forced vital capacity; FEV_1_: forced expiratory volume in the first second; FVC/FEV_1_: ratio of forced vital capacity to forced expiratory volume in the first second; FEF_25-75%_: forced expiratory flow at 25–75%; MIP: maximum inspiratory pressure; MEP: maximum expiratory pressure; SNIP: sniff nasal inspiratory pressure; SNEP: nasal expiratory pressure; m: meters; kg: kilograms; L: liters; %pred: percentage of predicted; L/s: liters for second; cmH_2_O: centimeters of water.

### RET parameters

Perceived exertion (p < 0.001) and HR (p < 0.001) significantly increased after both protocols, but no significant differences were observed between protocols. The duration of NH test tended to be longer than IPTL test (p = 0.07), and SpO_2_ at the end of NH test was lower than IPTL test (p = 0.008). Table 2 shows data regarding parameters, duration, vital signs, and perceived effort.

**Table 2:** Parameters from endurance test.

	IPTL	NH	p-value (Intragoup)	p-value (Intergroup)
Tlim (s)	151 ± 85.7	209.2 ± 119.6	-	0.07
Borg_initial_	0.63 ± 1.13	0.45 ± 1.0	-	0.61
Borg_Final_	6.68^a^ ± 1.78	7.13^a^ ± 1.32	<0.0001	0.39
HR_initial_	81.23 ± 9.86	76.32 ± 11.49	-	0.26
HR_Final_	104.9 ± 22.55	104.5 ± 21.49	<0.0001	0.62
SpO_2initial_ (%)	98 ± 0.87	98.3 ± 1.21	-	0.11
SpO_2Final_ (%)	98.6 ± 1.04	96.7 ± 3.45	<0.05	0.008
VE (l/min)	-	104.8 ± 18.88	-	-
RR	-	44.3 ± 7.79	-	-

Data presented as mean ± SD. Tlim: time to task failure; HRinitial: pre-test heart rate; HRfinal: post-test heart rate; SpO2initial: pre-test peripheral arterial saturation; SpO2Final: post-test peripheral arterial saturation; VE: minute volume; RR: respiratory rate; s: second; %: percentage; cmH_2_O: cen

### Respiratory endurance test

#### sEMG median frequency

**Fig 2:**
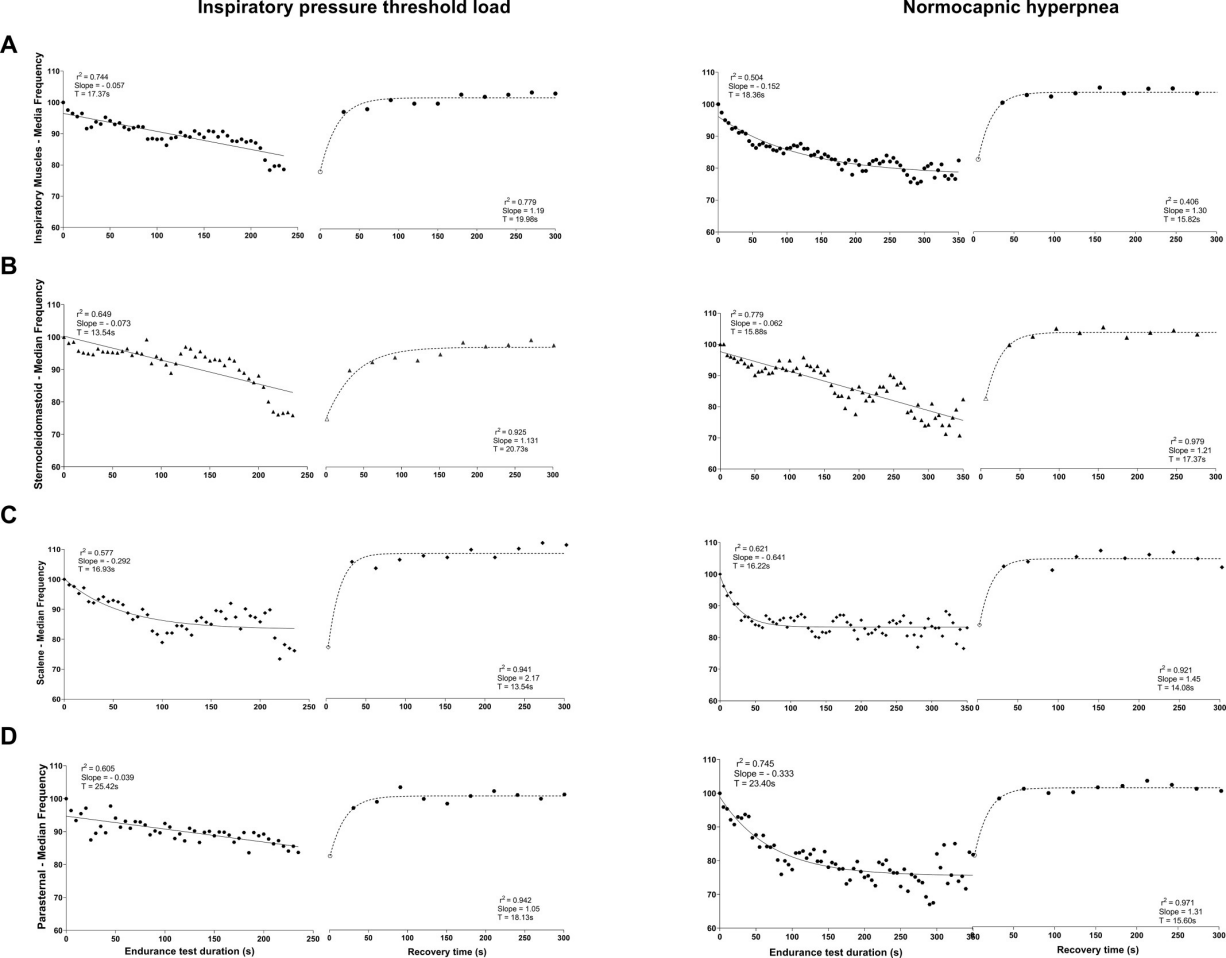
Time courses of normalized median frequency of inspiratory rib cage muscles. Mean of 22 subjects; inspiratory rib cage muscles (Panel A), sternocleidomastoid (Panel B), scalene (Panel C) and parasternal (Panel D) during endurance test and recovery. Each point during the test is the average of 5 seconds; during recovery, each point represents data extracted from one SNIP maneuver. In each muscle, starting point at time zero (white symbols) corresponds to the last point of endurance test.

The median frequency behaved differently between tests. A linear decrease in MF of inspiratory rib cage muscles was observed only during IPTL test, with SCM exhibiting the highest slope (slope = -0.073, r^2^ = 0.649) (Fig 2A). An exponential decrease was observed in MF of most muscles during NH test, except for SCM, which exhibited a linear decrease (slope = -0.062, r^2^ = 0.779) (Fig 2B). Inspiratory rib cage muscles showed a time constant (TF) of 17.37 s and 18.36 s in IPTL and NH test, respectively (Fig 2A). In both tests, SCM exhibited the lowest time constant value (TF = 13.54 s in IPTL and TF = 15.88 s in NH) (Fig 2B).

#### NIRS tissue oxygenation

**Fig 3:**
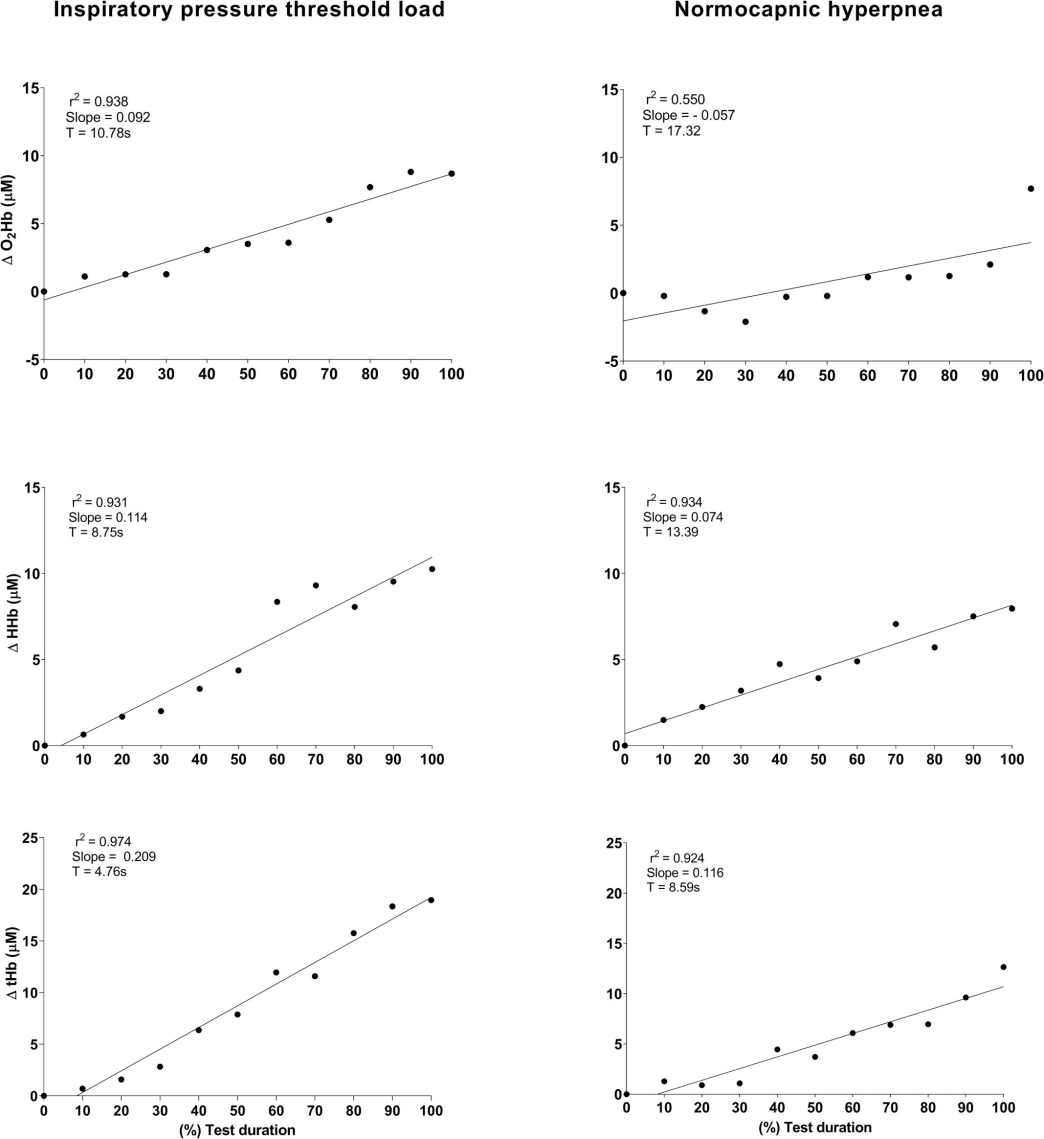
Time courses of tissue oxygenation variables of sternocleidomastoid muscle during endurance test. Each point during the test is the average of every 10% of test duration until time limit. **Abbreviations:** O2Hb = Oxyhemoglobin; HHb: deoxyhemoglobin; tHb: total hemoglobin. %: percentage.

The behavior of tissue oxygenation during RETs is shown in Fig 3. Each point represents the value of the variables analyzed at intervals of 10% of the total duration of each RET. O_2_Hb, HHb, and tHb increased linearly in both protocols, with tHb exhibiting the highest slope (slope = 0.209, r^2^ = 0.974; slope = 0.116, r^2^ = 0.924 − IPTL and NH, respectively). O_2_Hb exhibited the lowest slope values (slope = 0.092, r^2^ = 0.938; slope = 0.057, r^2^ = 0.550 − IPTL and NH, respectively) (Fig 3).

### Recovery phase

#### SNIP contractile and relaxation parameters

**Fig 4:**
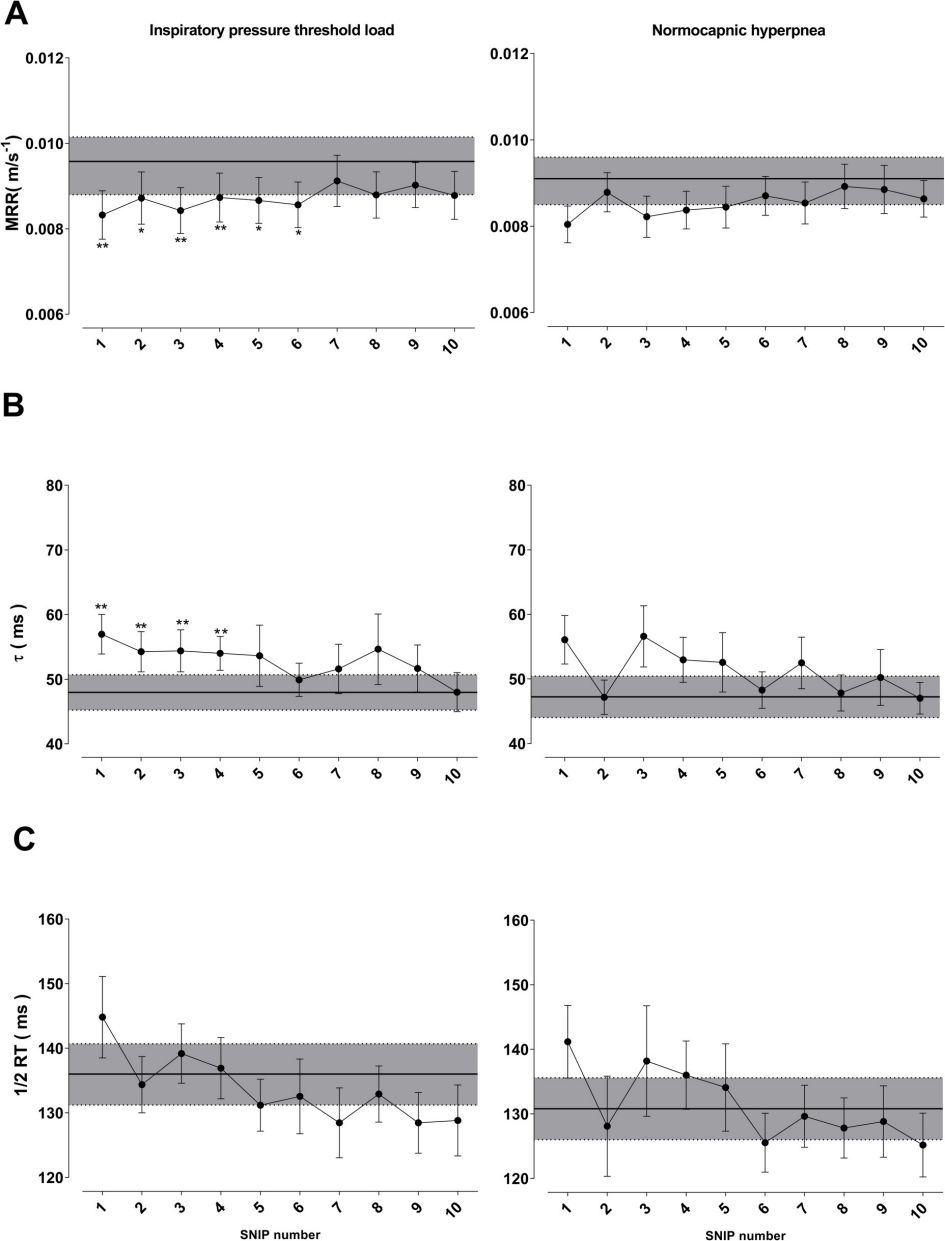
Changes in maximum relaxation rate (MRR-panel A), time constant (tau) of relaxation curve (τ-panel B), and time to reach half of relaxation curve (½ RT-panel C). Comparison of values during recovery from endurance test and pre-test (gray band). Data presented as mean ± SE. **Abbreviation:** ms: milliseconds. *Statistically significant intragroup difference between pre-test and recovery p <0.05 **Statistically significant intragroup difference between pre-test and recovery p <0.01.

**Fig 5:**
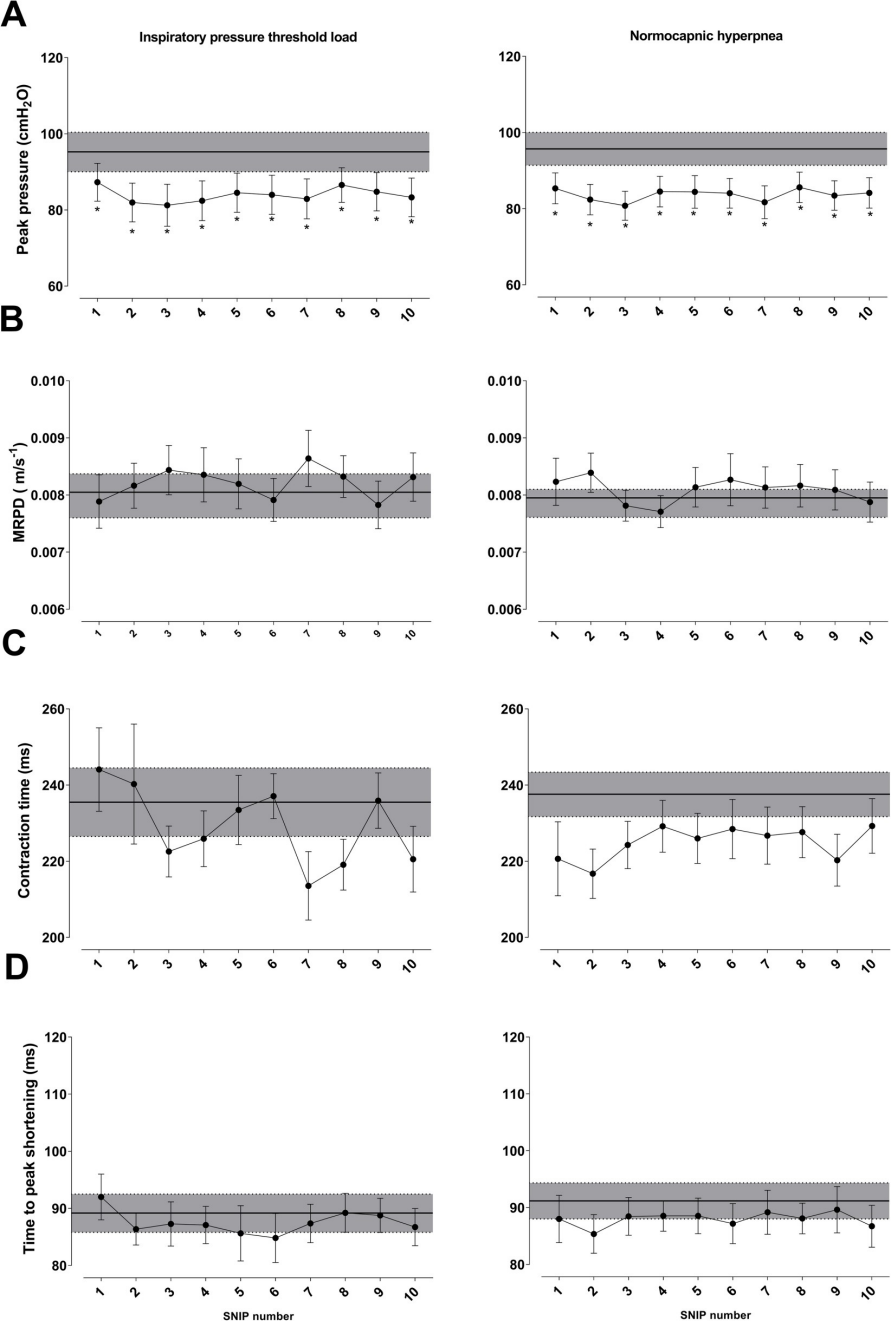
Changes in peak pressure of SNIP (panel A), maximum rate of pressure development (MRPD-panel B), contraction time (CT-panel C), and time to reach MRPD (TPS-panel D). Comparison of values during recovery from endurance test and pre-test (gray band). Data presented as mean ± SE. **Abbreviations:** cmH_2_O: centimeters of water; ms: milliseconds. *Statistically significant intragroup difference between pre-test and recovery p <0.05.

Data regarding SNIP contractile and relaxation parameters from the pre-RET maneuver and 10 post-RET maneuvers are shown in Figs 4 and 5. Relaxation parameters exhibited a statistically significant difference only in intragroup comparisons of IPTL test. MRR significantly decreased from the first to sixth post-RET maneuver (p < 0.05), whereas τ significantly increased from the first to fourth maneuver (p < 0.01), returning to baseline values in the tenth maneuver (Fig 4).

Peak pressure values generated during all SNIP maneuvers after RET were lower than pre-RET values in both protocols (p < 0.05) (Fig 5). MRPD, TPS, and CT were not significantly different between moments and RETs.

### OEP shortening velocity index and mechanical power

**Fig 6:**
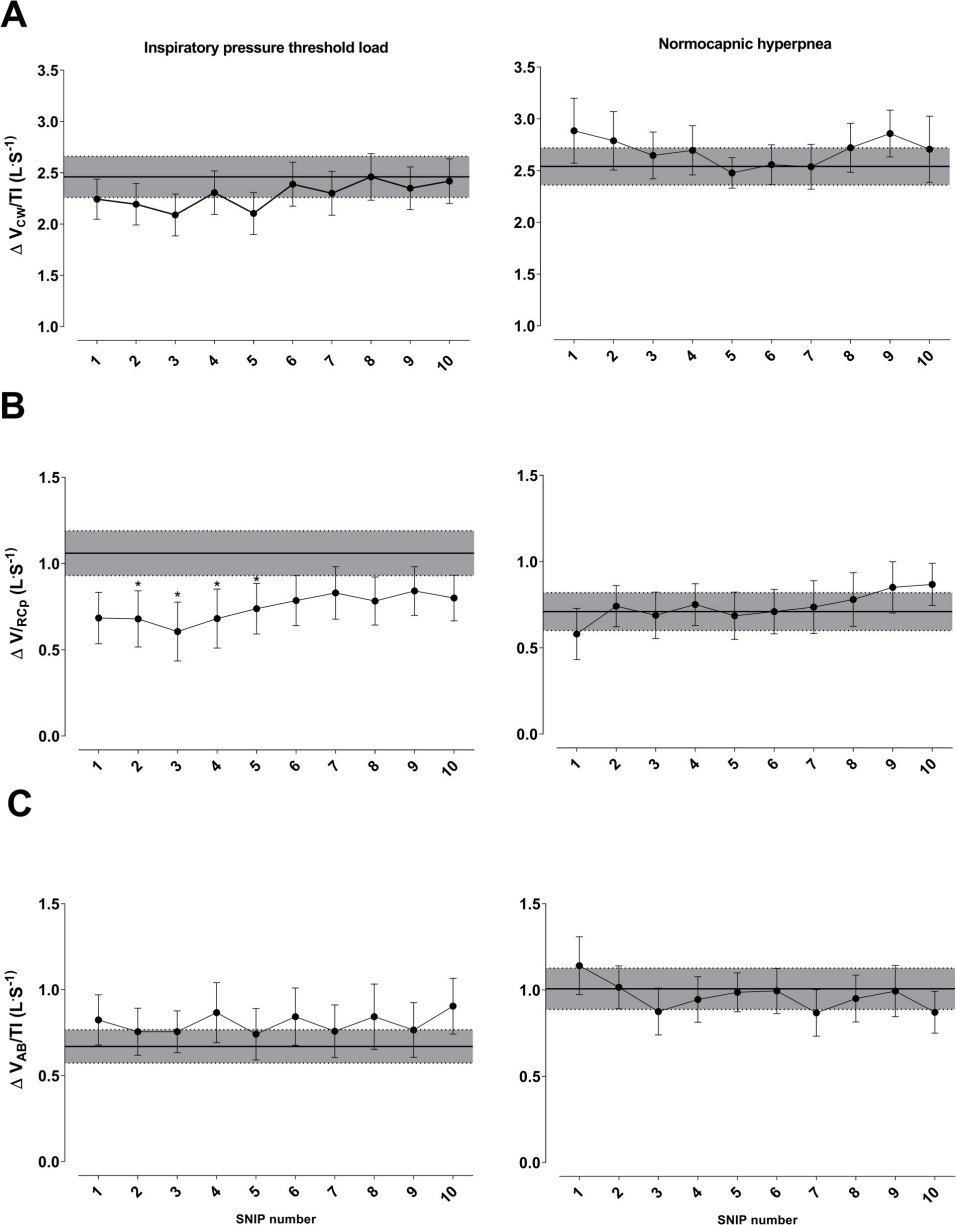
Changes in shortening velocity of global inspiratory muscles (ΔV_CW_ / Ti- panel A), inspiratory rib cage muscles (ΔV_RCp_ / Ti- panel B), and diaphragm (ΔV_AB_ / Ti- panel C). Comparison of values during recovery of endurance test and pre-test (gray band). Data presented as mean ± SE. **Abbreviation:** L.s^-1^: liters per second. *Statistically significant intragroup difference between pre-test and recovery p <0.05.

**Fig 7:**
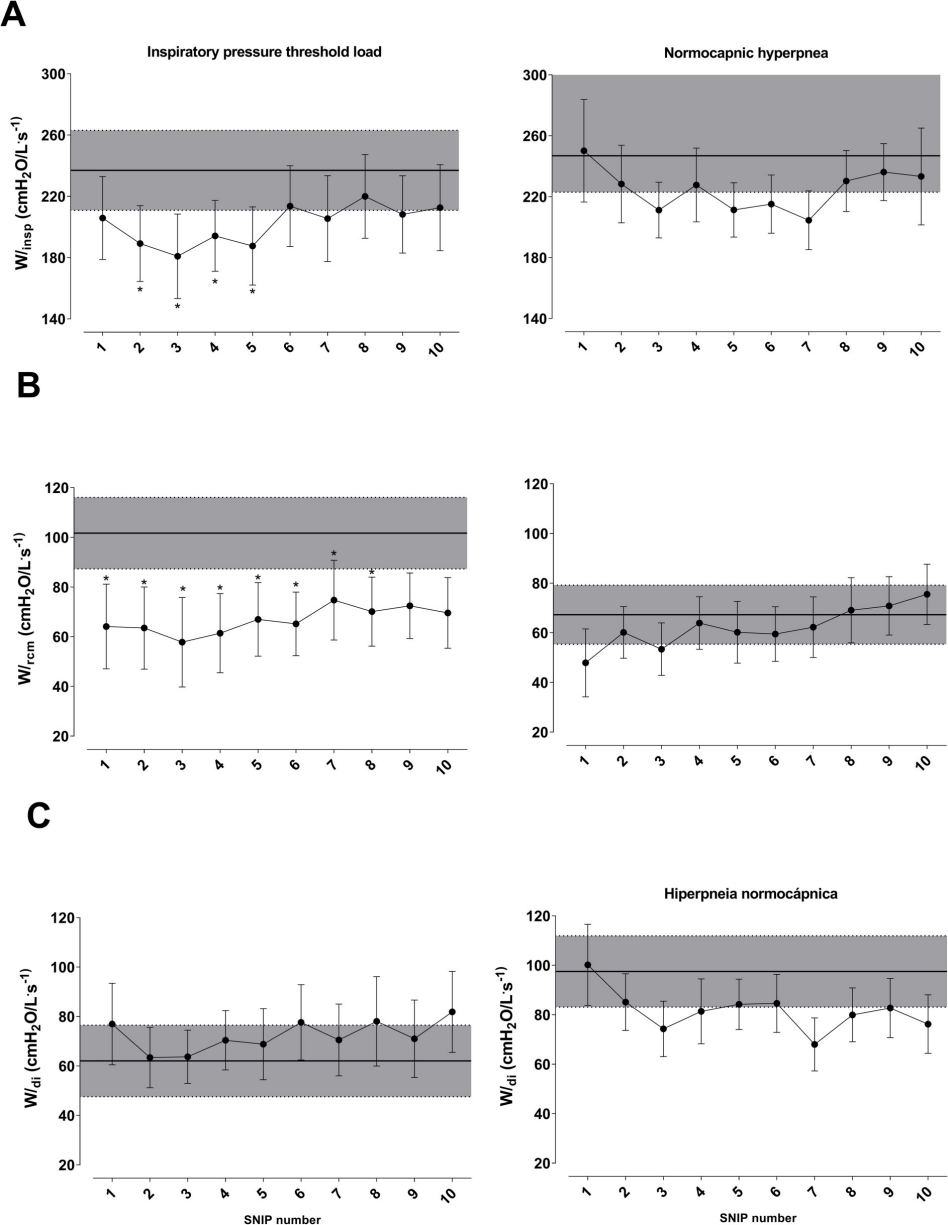
Changes in mechanical power of global inspiratory muscles (Winsp−panel A), inspiratory rib cage muscles (W_rcm−_panel B), and diaphragm (Wdi−panel C). Comparison of values during recovery of endurance test and pre-test (gray band). Data presented as mean ± SE. **Abbreviations:** cmH_2_O: centimeters of water; L.s: liters per second. *Statistically significant intragroup difference between pre-test and recovery. p <0.05.

Data regarding shortening velocity and mechanical power obtained from OEP are shown in Figs 6 and 7. These figures represent the values obtained during the 10 maneuvers in the recovery phase (post-RET) and the pre-RET maneuver. Shortening velocity and mechanical power were different only in the IPTL test. ΔV_RCp_/Ti decreased from the second to fifth post-RET maneuver (p < 0.05) after IPTL test. ΔV_CW_/Ti and ΔV_AB_/Ti post-RET were not different compared with pre-RET values. No significant intergroup changes were observed in the NH test (Fig 6).

W_insp_ decreased from the second to fifth maneuver in the IPTL test (p < 0.05), whereas W_rcm_ decreased from the first to eighth maneuver (p < 0.05) (Fig 7).

### sEMG muscle electrical activity

**Fig 8:**
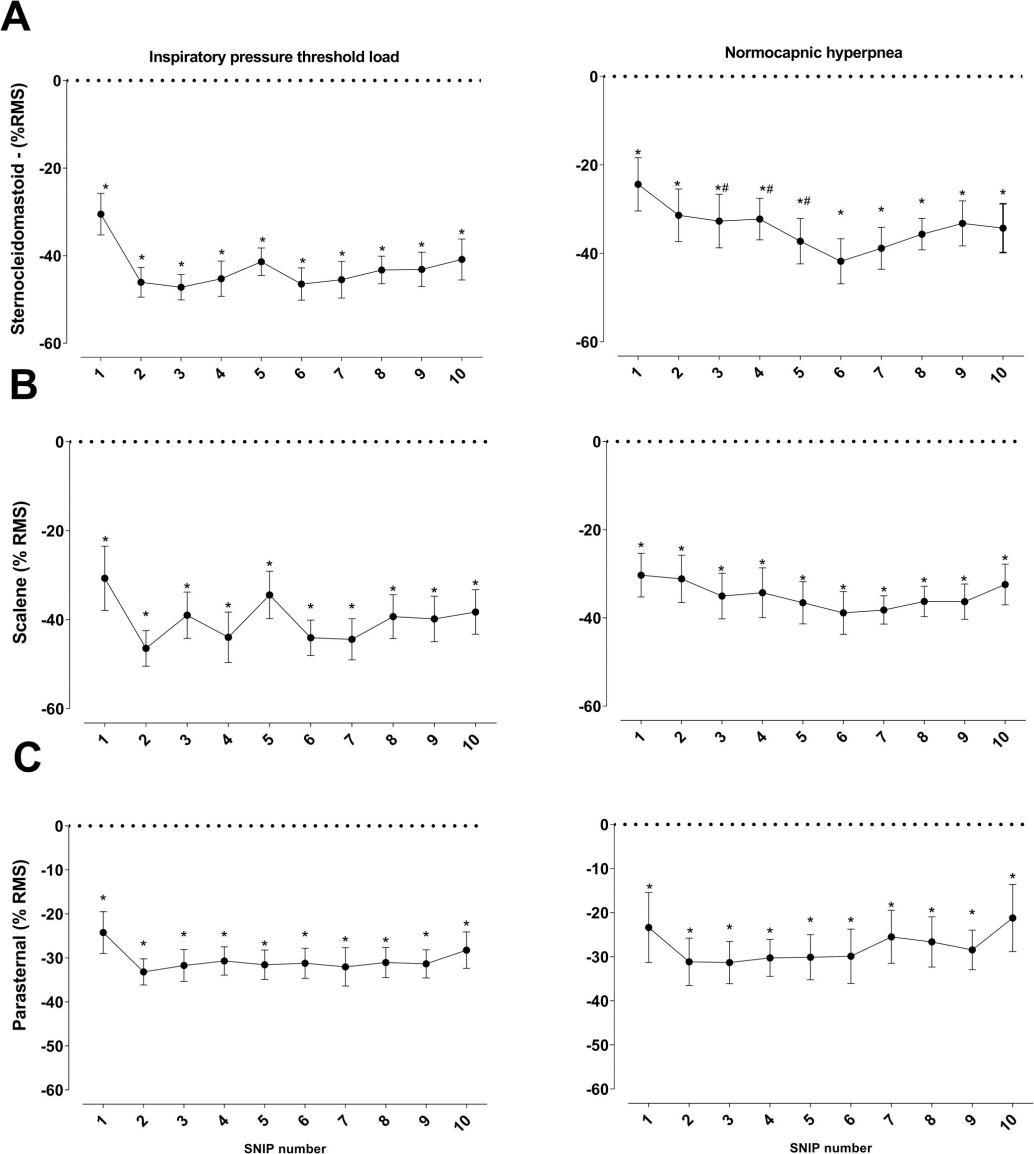
Changes in activation (RMS) of sternocleidomastoid (panel A), scalene (panel B), and parasternal muscles (panel C) compared with pre-test values. Data presented as mean ± SE. *p <0.05 #: intergroup difference (p <0.05).

Fig 8 shows data on inspiratory muscle electrical activity (RMS) obtained during the 10 maneuvers in the recovery phase (post-RET), which were analyzed as percentage of pre-RET value. Similar muscle activation patterns were observed in SCM, ESC, and PS muscles in both tests, with significant decrease (p < 0.05) in %RMS in all post-RET moments compared with pre-RET. Electrical activity of SCM decreased significantly from the second to fourth maneuver (p < 0.05) in the IPTL test compared with NH test (Fig 8). During recovery of MF, Trec values of 19.98 and 15.82 s were observed in IPTL and NH test, respectively. SCM exhibited a longer recovery time than other muscles in both tests (Trec = 20.73 s in IPTL and Trec = 17.37 s in NH) (Fig 2).

## Discussion

### Main findings

The IPTL protocol with 80% of MIP resulted in a linear decrease of MF and statistically significant differences in relaxation properties (MRR and τ), peak pressure, shortening velocity, and mechanical power of inspiratory rib cage muscles between post-RET and pre-RET. Concentrations of O_2_Hb, HHb, and tHb in the SCM increased during both RET protocols.

### Inspiratory rib cage muscle fatigue

Inspiratory muscle fatigue has been assessed using the duration of sustained load or volume, maximum respiratory pressures, or by assessing dyspnea and perceived effort [29]. As these variables are subjective and imprecise to analyze fatigue development [47], we used the frequency parameters of sEMG to analyze fatigue development. Studies showed decreased MF as function of time during fatigue by assessing the slope generated during a particular test (dynamic or isometric) [48–50]. After analyzing each muscle individually, SCM exhibited the greatest negative slope during IPTL test. This finding was probably due to the higher proportion of type II fibers in the SCM (65%) than ESC and PS (39% and 38%, respectively) [51, 52], which may also explain the linear slope during NH protocol, the short TF, and long Trec observed in the SCM compared with other muscles in both protocols.

Although MF of inspiratory rib cage muscles decreased exponentially during the NH protocol, it did not reach the lower limit used to characterize fatigue (60%). Many studies assessing the development of inspiratory muscle fatigue during NH protocol analyzed diaphragm fatigue using invasive techniques [1, 29, 53, 54]. Renggli et al. performed an NH protocol in healthy individuals and observed fatigue in the diaphragm and rectus abdominis before task failure. Moreover, the RET time was higher than in the present study, and increased recruitment of inspiratory and expiratory muscles maintained high ventilatory levels [29]. Therefore, the recruitment of expiratory muscles and short duration of RET occurred probably because the NH protocol did not reach significant levels for fatigue development.

The MF of inspiratory rib cage muscles decreased linearly during IPTL test. This finding corroborates Sarmento et al. [8], who performed a RET protocol similar to the one used in this study and observed decreased MF of inspiratory rib cage muscles. Slopes of MF obtained in our study were smaller than those obtained by Sarmento et al. [8], probably because of different loads used (80% vs. 70% of MIP). Therefore, differences in MF behavior between NH and IPTL may also be associated with pattern of muscle recruitment and structural adaptations occurring during each test [2].

### Tissue oxygenation during RET

Inspiratory muscle fatigue increases sympathetic nerve activity because this response is mediated mainly by type III and IV afferent fibers from diaphragm [5–7, 55]. This process is called inspiratory muscle metaboreflex, and when activated, blood flow is reduced to inactive muscles and redirected to active muscles. Shadgan et al. [43] and Basoudan et al. [56] observed increased HHb, tHb, and O_2_Hb in the SCM during inspiratory resistive load. Guenette et al. [57] observed increased blood flow to respiratory muscles with enhanced ventilatory work during NH protocol. Our findings were similar to those studies, with increased tHb as function of time, probably due to the redirection of blood flow from inactive muscles to respiratory muscles during activity. However, local blood flow of peripheral muscle was not estimated in the present study. The increased HHb and O_2_Hb can be explained by the Bohr effect, which results in oxygen discharge due to acidity from increased activity and temperature [43]. Moreover, the increase in the estimated blood flow (tHb) to the SCM during RET also may explain the maintenance of O_2_Hb levels, hypothesizing that the delivery of blood supply was sufficient to maintain O_2_Hb even with an increase in HHb. Regardless of the protocol, enhanced respiratory work changed blood flow and tissue oxygenation.

### Recovery phase (post-RET)

#### Contractile and relaxation parameters

Previous studies showed SNIP maneuver was correlated with esophageal pressure, diaphragmatic pressure, and strength other inspiratory muscles [15]. Previous reports also suggested fatigue changes relaxation properties, such as MRR and τ [11, 15, 16]. Mador et al. [16] observed decreased MRR and increased τ in healthy individuals after fatigue using an inspiratory load. The present study showed similar results in MRR and τ only after IPTL, and values returned to baseline in the seventh and tenth maneuvers, respectively. This result may be associated with changes in sarcoplasmic reticulum due to fatigue, leading to changes in calcium release and uptake capacity. Additionally, the accumulation of phosphorus during fatigue development may have altered maximum relaxation rates [58–60].

#### Shortening velocity index and mechanical power

Loss of power during muscle fatigue can occur due to changes in strength, shortening velocity, or both. We observed a decreased shortening velocity (ΔV_RCp_) and mechanical power (W_insp_ and W_rcm_) only after the IPTL protocol, probably because of reduced force and shortening velocity of chest wall inspiratory muscles [8]. These alterations occur due to metabolic changes induced by fatigue, affecting cross-bridges and actin-myosin binding, hindering the formation of a new coupling-uncoupling cycle, and reducing muscle contraction [61].

#### Muscle electrical activity

The electrical activity of inspiratory ribc age muscles was similar in both protocols, with reduced %RMS in post-RET compared with pre-RET maneuvers. In the intergroup comparison, the %RMS of SCM was lower in the IPTL test, probably due to the pattern of muscle recruitment during RET. For example, the negative slope of SCM during the IPTL protocol may have potentiated fatigue development due to greater recruitment. Decreased muscle electrical activity due to increased respiratory effort may be associated with decreased conduction velocity of action potential, reducing the electrical excitation of the muscle [62, 63].

#### Practical applications

Based on our results, non-invasive measures used in this study may be an alternative for monitoring inspiratory rib cage muscle fatigue in healthy individuals. Low-cost and easy to obtain measures, such as relaxation parameters from SNIP maneuver, can also be performed in medical clinics to assess individuals with ventilatory failure. In our study, perceived effort and time were not different between RET modalities, suggesting equivalent tolerance and adherence. In this sense, tests can be chosen according to muscles and recruitment patterns since IPTL involves high load and components related to strength, whereas NH is characterized by low load and endurance components [2].

#### Strengths and limitations of the study

This is the first study to assess the physiological behavior of respiratory muscles before, during, and after different respiratory endurance tests using simultaneous instruments (e.g., OEP, sEMG, and NIRS). Inspiratory muscle fatigue is present in several diseases; therefore, understanding the physiological changes caused by fatigue development in inspiratory rib cage muscles during different RET help decide the best method to assess fatigue in individuals with reduced resistance to fatigue.

The present study has some limitations. The high percentage of MIP during the IPTL protocol may have led to shorter test duration, limiting the potentiation of fatigue development. We did not use another NIRS probe to assess peripheral muscle oxygenation and evidence inspiratory metaboreflex, and sEMG of rectus abdominis was not performed. Hence, questions regarding different recruitments between protocols could not be elucidated.

## Conclusion

The behavior of inspiratory rib cage muscles of healthy individuals differs between NH and IPTL endurance tests. Changes in relaxation rates (MRR and τ), shortening velocity, and mechanical power were observed only after IPTL test. Fatigue development in inspiratory rib cage muscles and consequent changes may be more evident after IPTL test, possibly because of increased recruitment of inspiratory rib cage muscles. In general, inspiratory muscle fatigue changes the behavior of inspiratory rib cage muscles.

## Supporting information

S1 Appendix Calculation of slopes of exponential regression lines. (DOCX)

S1 Data (XLSX)

## References

[1] Kabitz H.J., Walker D.J., Schwoerer A., Schlager D., Walterspacher S., Storre J.H., et al. Biometric approximation of diaphragmatic contractility during sustained hyperpnea. Respir Physiol Neurobiol. May 2011. 31;17, 176 (3), 90–7. doi: 10.1016/j.resp.2011.01.011 21295161

[2] Verges S., Renggli A.S., Notter D.A., Spengler C.M. Effects of different respiratory muscle training regimes on fatigue-related variables during volitional hyperpnoea. Respir Physiol Neurobiol. Dec 2009 31:169(3),282–90. doi: 10.1016/j.resp.2009.09.005 19761874

[3] Laghi F., Topeli A., Tobin M.J. Does resistive loading decrease diaphragmatic contractility before task failure? J Appl Physiol. Sep 1998 85(3),1103–12. doi: 10.1152/jappl.1998.85.3.1103 9729589

[4] Walker D.J., Farquharson F., Klenze H., Walterspacher S., Storz L., Duerschmied D, et al. Diaphragmatic fatigue during inspiratory muscle loading in normoxia and hypoxia. Respir Physiol Neurobiol. Jun 2016 15:227,1–8, 20. doi: 10.1016/j.resp.2016.01.006 26845453

[5] Gibson G.J., Whitelaw W., Siafakas N., Supinski G.S., Fitting J.W., Bellemare F., et al. ATS/ERS Statement on respiratory muscle testing. Am J Respir Crit Care Med. Aug 2002 15:166, 518–624. doi: 10.1164/rccm.166.4.518 12186831

[6] Klimathianaki M., Vaporidi K., Georgopoulos D. Respiratory muscle dysfunction in COPD: from muscles to cell. Curr Drug Targets. Apr 2011 12(4),478–88. doi: 10.2174/138945011794751474 21194407

[7] Katayama K., Iwamoto E., Ishida K., Koike T., Saito M. Inspiratory muscle fatigue increases sympathetic vasomotor outflow and blood pressure during submaximal exercise. Am J Physiol. May 2012 15;302(10),1167–75. doi: 10.1152/ajpregu.00006.2012 22461178

[8] Sarmento A., Fregonezi G., Lira M., Marques L., Pennati F., Resqueti A., et al. Changes in electromyography activity, mechanical power, and relaxation rates following inspiratory ribcage muscle fatigue. Sci Rep. Jun 2021 14;11(1). 10.1038/s41598-021-92060-y. 341sar754 PMC8203654 34127754

[9] Jewell B.R., Wilkie D.R. The mechanical properties of relaxing muscle. J Physiol. Jun 1960 152(1),30–47. doi: 10.1113/jphysiol.1960.sp006467 14407245 PMC1363293

[10] Edwards R.H., Hill D.K., Jones D.A. Metabolic changes associated with the slowing of relaxation fatigued mouse muscle. J Physiol. Oct 1975 251(2),287–301. doi: 10.1113/jphysiol.1975.sp011093 1185665 PMC1348428

[11] Kyroussis D., Mills G., Hamnegard C.H, Road J., Green M., Wragg S., et al. Inspiratory muscle relaxation rate assessed from sniff nasal pressure. Thorax. Nov 1994 49(11),1127–33. doi: 10.1136/thx.49.11.1127 7831629 PMC475274

[12] Romer L.M., McConnell A.K. Inter-test reliability for non-invasive measures of respiratory muscle function in healthy humans. Eur J Appl Physiol. Mar 2004 91(2–3),167–76. doi: 10.1007/s00421-003-0984-2 14605897

[13] Koulouris N., Vianna L.G., Mulvey D.A., Green M., Moxham J. Maximal relaxation rates of esophageal, nose, and mouth pressures during a sniff reflect inspiratory muscle fatigue. Am Rev Respir Dis. May 1989 139(5),1213–7. doi: 10.1164/ajrccm/139.5.1213 2712448

[14] Dassios T.G., Doudounakis S., Dimitriou G. Maximum Rate of Pressure Development and Maximal Relaxation Rate of Respiratory Muscles in Patients with Cystic Fibrosis. Respir Care. Mar 2013 58(3),474–81. doi: 10.4187/respcare.01930 22781492

[15] Esau S.A., Bellemare F., Grassino A., Permutt S., Roussos C., Pardy R.L. Changes in relaxation rate with diaphragmatic fatigue in humans. J Appl Physiol. May 1983 54(5),1353–60. doi: 10.1152/jappl.1983.54.5.1353 6863095

[16] Mador M.J, Kufel T.J. Effect of inspiratory muscle fatigue on inspiratory muscle relaxation rates in healthy subjects. Chest. Dec 1992 102(6),1767–73. doi: 10.1378/chest.102.6.1767 1446487

[17] Sarmento A., Aliverti A., Marques L., Pennati F., Dourado-Júnior M.E., Fregonezi G., et al. Multiparametric analysis of sniffnasal inspiratory pressure test in middle stage amyotrophic lateral sclerosis. Front Neurol. May 2018 2, 9:306 doi: 10.3389/fneur.2018.00306 29770120 PMC5940741

[18] Beck T.W., Stock M.S., De Freitas J.M. Shifts in EMG spectral power during fatiguing dynamic contractions. Muscle Nerve. Jul 2014 50(1),95–102. doi: 10.1002/mus.24098 24122808

[19] de Luca C.J. The use of surface electromyography in biomechanics. J Appl Biomech. 1997 13,135–63.

[20] Gallagher C.G., Hof V.I., Younes M. Effect of inspiratory muscle fatigue on breathing pattern. J Appl Physiol. Oct 1985 59(4), 1152–8. doi: 10.1152/jappl.1985.59.4.1152 4055595

[21] Verges S., Notter D., Splenger C.M. Influence of diaphragm and rib cage muscle fatigue on breathing during endurance exercise. Respir Physiol Neurobiol. Dec 2006 154(3), 431–42. doi: 10.1016/j.resp.2005.12.007 16423567

[22] Illi S.K., Hostettler S., Aliverti A., Spengler C.M. Compartmental chest wall volume changes during volitional hyperpnoea with constant tidal volume in healthy individuals. Resp Physiol Neurobio. Jan 2013 185(2), 410–5. doi: 10.1016/j.resp.2012.08.018 22959999

[23] Hostettler S., Illi S.K., Mohler E., Aliverti A., Spengler C.M. Chest wall volume changes during inspiratory loaded breathing. Resp Physiol Neurobiol. Jan 2011 175(1),130–9. doi: 10.1016/j.resp.2010.10.001 20937414

[24] Aliverti A., Ghidoli G., Dellaca R.L., Pedotti A., Macklem P.T. Chest wall kinematic determinants of diaphragm length by optoelectronic plethysmography and ultrasonography. J Appl Physiol. Feb 2003 94 (2), 621–630. doi: 10.1152/japplphysiol.00329.2002 12391129

[25] Aliverti A., Iandelli I., Duranti R., Cala S.J., Kayser B., Kelly S., et al. Respiratory muscle dynamics and control during exercise with externally imposed expiratory flow limitation. J Appl Physiol. May 2002 92(5), 1953–1963. doi: 10.1152/japplphysiol.01222.2000 11960945

[26] Adami A., Cao R., Porszasz J., Casaburi R., Rossiter H.B. Reproducibility of NIRS assessment of muscle oxidative capacity in smokers with and without COPD. Respir Physiol Neurobiol. Jan 2017 235,18–26. doi: 10.1016/j.resp.2016.09.008 27659351 PMC5136338

[27] Tanaka T., Basoudan N., Melo L.T., Wickerson L., Brochard L.J., Goligher E.C., et al. Deoxygenation of inspiratory muscles during cycling, hyperpnoea and loaded breathing in health and disease: a systematic review. Clin Physiol Funct Imaging. Jul 2018 38(4), 554–65. doi: 10.1111/cpf.12473 28940670

[28] Pardini R., Matsudo S.M., Araújo T., Matsudo V., Andrade E., Braggion G., et al. Validação do questionário internacional de nível de atividade física (IPAQ–versão 6): estudo piloto em adultos jovens brasileiros. Rev. Bras. Ciên. 2001.

[29] Renggli A.S., Verges S., Notter D.A., Spengler C.M. Development of respiratory muscle contractile fatigue in the course of hyperpnoea. Respir Physiol Neurobiol. Dec 2008 164(3),366–72. doi: 10.1016/j.resp.2008.08.008 18801466

[30] Janssens L., Brumagne S., Mcconnell A.K., Raymaekers J., Goossens N., Gayan-ramirez G. The assessment of inspiratory muscle fatigue in healthy individuals: A systematic review. Respir Med. Marc 2013 107(3):331–46. doi: 10.1016/j.rmed.2012.11.019 23273596

[31] Moxham J., Morris A.J., Spiro S.G., Edwards R.H., and Green M. Contractile properties and fatigue of the diaphragm in man. Thorax. Mar 1981 36(3), 164–168. doi: 10.1136/thx.36.3.164 7281080 PMC471468

[32] Miller M.R., Hankinson J., Brusasco V., Burgos F., Casaburi R., Coates A., et al. J ATS/ERS Task Force. Standardisation of spirometry. Eur Respir J. Aug 2005 26 (2),319–38. doi: 10.1183/09031936.05.00034805 16055882

[33] Pereira C.A, Sato T., Rodrigues S.C. Novos valores de referência para espirometria forçada em brasileiros adultos de raça branca. Jornal Brasileiro de Pneumologia 2007 33,97–406. 10.1590/S1806-37132007000400008

[34] Neder J.A., Andreoni S., Lerario M.C., Nery L.E. Reference values for lung function tests. II. Maximal respiratory pressures and voluntary ventilation. Braz J Med Biol Res. Jun 1999 32(6),719–27. doi: 10.1590/s0100-879x1999000600007 10412550

[35] Araújo P.R.S., Resqueti V.R., Nascimento J. Jr., et al. Valores de referência da pressão inspiratória nasal em indivíduos saudáveis no Brasil: estudo multicêntrico. Jornal Brasileiro de Pneumologia. 2012 38,700–7. 10.1590/S1806-37132012000600004 23288114

[36] Metzger J.M., Moss R.L. Shortening velocity in skinned single muscle fibers. Influence of filament lattice spacing. Biophys J. Jul 1987 52(1),127–31. doi: 10.1016/S0006-3495(87)83197-1 3607220 PMC1329992

[37] Herve P., Lecarpentier Y., Brenot F., Clergue M., Chemla D., Duroux P. Relaxation of the diaphragm muscle: influence of ryanodine and fatigue. J Appl Physiol. Nov 1988 65(5),950–6. doi: 10.1152/jappl.1988.65.5.1950 3209544

[38] Aliverti A., Cala S.J., Duranti R., Ferrigno G., Kenyon C.M., Pedotti A., et al. Human respiratory muscle actions and control during exercise. J Appl Physiol. Oct 1997 83(4),1256–69. doi: 10.1152/jappl.1997.83.4.1256 9338435

[39] Hermens H.J., Freriks B., Disselhorst-Klug C.R.G. Development of recommendations for SEMG sensors and sensor placement procedures. J Electromyogr Kinesiol. Oct 2000 10(5):361–74. doi: 10.1016/s1050-6411(00)00027-4 11018445

[40] De Andrade A.D., Silva T.N., Vasconcelos H., Marcelino M., Rodrigues-Machado M.G., Filho V.C., et al. Inspiratory muscular activation during threshold therapy in elderly healthy and patients with COPD. J Electromyogr Kinesiol. Dec 2005 15(6),631–9. doi: 10.1016/j.jelekin.2005.06.002 16051499

[41] Da Cunha A.P.N.M. Silva T.N.S., França E.R.T., Amorim C., Galindo Filho V.C., De Andrade A.D. Efeito do alongamento sobre a atividade dos músculos inspiratórios na DPOC. 2005 7:6.

[42] Duiverman M.L., van Eykern L.A., Vennik P.W., Koeter G.H., Maarsingh E.J., Wijkstra P.J. Reproducibility and responsiveness of a noninvasive EMG technique of the respiratory muscles in COPD patients and in healthy subjects. J Appl Physiol. May 2004 96 (5),1723–9. doi: 10.1152/japplphysiol.00914.2003 14660508

[43] Shadgan B., Guenette J.A., Sheel A.W., Reid W.D. Sternocleidomastoid muscle deoxygenation in response to incremental inspiratory threshold loading measured by near infrared spectroscopy. Respir Physiol Neurobiol. Sep 2011 178(2),202–9. doi: 10.1016/j.resp.2011.06.001 21684356

[44] Benjamini Y., Krieger A.M., and Yekutieli D. Adaptive linear step-up procedures that control the false discovery rate. Biometrika. 2006 93,491–507.

[45] Cifrek M., Tonković S., and Medved V. Measurement and analysis of surface myoelectric signals during fatigued cyclic dynamic contractions. Measurement. 2000 27,85–92. 10.1016/S0263-2241(99)00059-7

[46] Aubier M., Farkas G., De Troyer A., Mozes R., and Roussos C. Detection of diaphragmatic fatigue in man by phrenic stimulation. J Appl Physiol. Mar 1981 50(3),538–544. doi: 10.1152/jappl.1981.50.3.538 7251445

[47] Grassino A., M D., Macklem P.T. Respiratory muscle fatigue and ventilatory failure. Chest. 1990 97,89–96. 10.1378/chest.97.3_Supplement.89S 2155091

[48] Gertler J., Cao J. EMG spectral power and fatigue. Wiley Intersci. 2004 50,1–15.

[49] Merletti R., Knaflitz M., De Luca C.J. Myoelectric manifestations of fatigue in voluntary and electrically elicited contractions. J Appl Physiol. Nov 1990 69(5),1810–20. doi: 10.1152/jappl.1990.69.5.1810 2272975

[50] Komi P. V., Tesch P., Komi V., Tesch P. EMG Frequency Spectrum, Muscle Structure and Fatigue. Eur J Appl Physiol. Sep 1979 42(1),41–50. doi: 10.1007/BF00421103 499196

[51] Johnson M.A., Polgar J., Weightman D., and Appleton D. Data on the distribution of fibre types in thirty-six human muscles. An autopsy study. Journal of the neurological sciences. Jan 1973 18(1),111–129. doi: 10.1016/0022-510x(73)90023-3 4120482

[52] Vikne H., Gundersen K., Liestol K., Maelen J., and Vollestad N. Intermuscular relationship of human muscle fiber type proportions: slow leg muscles predict slow neck muscles. Muscle & nerve. Apr 2012 45(4),527–535. doi: 10.1002/mus.22315 22431086

[53] Bai T.R., Rabinovitch B.J., Pardy R.L. Near-maximal voluntary hyperpnea and ventilatory muscle function. J Appl Physiol Respir Environ Exerc Physiol. Dec 1985 57(6):1742–8. 10.1152/jappl.1984.57.6.1742. 6511549

[54] Verges S., Bachasson D., Wuyam B. Effect of acute hypoxia on respiratory muscle fatigue in healthy human. Respir Res. Aug 2010 11(1),109. doi: 10.1186/1465-9921-11-109 20701769 PMC2929221

[55] Sheel A.W., Boushel R., Dempsey J.A. Competition for blood flow distribution between respiratory and locomotor muscles: Implications for muscle fatigue. J Appl Physiol. Sep 2018 125(3),820–31. doi: 10.1152/japplphysiol.00189.2018 29878876 PMC6842878

[56] Basoudan N., Shadgan B., Guenette J.A., Road J., Reid W.D. Effect of acute hypoxia on inspiratory muscle oxygenation during incremental inspiratory load in healthy adults. Eur J Appl Physiol. Apr 2016 116(4),841–850. doi: 10.1007/s00421-016-3334-x 26892509

[57] Guenette J.A., Vogiatzis I., Zakynthinos S., et al. Human respiratory muscle blood flow measured by near-infrared spectroscopy and indocyanine green. J Appl Physiol. Apr 2008 104(4),1202–1210. doi: 10.1152/japplphysiol.01160.2007 18218914

[58] Allen D.G., Lãnnergren J., Westerblad H. Muscle Cell Function During Prolonged Activity: Cellular Mechanisms of Fatigue. Exp Physiol. Jul 1995 80(4),497–527. doi: 10.1113/expphysiol.1995.sp003864 7576593

[59] Favero T.G. Sarcoplasmic reticulum Ca2+ release and muscle fatigue. J Appl Physiol. Aug 1999 87,471–83. doi: 10.1152/jappl.1999.87.2.471 10444601

[60] Westerblad H., Allen D.G. Cellular mechanisms of skeletal muscle fatigue. Adv Exp Med Biol. 2003 538,563–70. doi: 10.1007/978-1-4419-9029-7_50 15098699

[61] Fitts R.H. The cross-bridge cycle and skeletal muscle fatigue. J Appl Physiol. Feb 2008 104(2),551–8. doi: 10.1152/japplphysiol.01200.2007 18162480

[62] Bigland-Ritchie B., Johansson R., Lippold O.C., Woods J.J. Contractile speed and EMG changes during fatigue of sustained maximal voluntary contractions. J Neurophysiol. Jul 1983 50(1),313–24. doi: 10.1152/jn.1983.50.1.313 6308182

[63] Sadoyama T., Miyano H., Higashi Y. Frequency Analysis of Surface EMG to Evaluation of Muscle Fatigue. Eur J Appl Physiol. 1981 47,239–46. doi: 10.1007/BF00422469 7198034

