# Supplementary material for: Non-invasive assessment of fatigue and recovery of inspiratory rib cage muscles during endurance test in healthy individuals
Source: PLoS One. 2022 Dec 7;17(12):e0277131. doi: 10.1371/journal.pone.0277131 (PMC9728934; doi:10.1371/journal.pone.0277131)
Supplement: S1 Appendix — (DOCX) [file pone.0277131.s001.docx]

**Appendix**

Calculation of slopes of exponential regression lines

All exponential curves that fitted values of present study reached a plateau. In this sense, equation best describing this behavior during fatigue protocol (decay) is expressed as follows:

y = (y_0_ - a) e^-k·t^ + a (E1)

Where “y” is any value in y-axis; “t” is time in x-axis; “y_0_” is value in y-axis when t equals “zero”; “a” is value of y-axis at high t values (asymptotic value); “k” is rate constant value expressed as inverse value of x-axis.

To allow comparisons between slopes of linear and exponential regressions, time derivative of equation E1 was calculated at the beginning of fatigue protocol (t = 0) as follows:

_dy/dt_|_t=0_ = -k (y_0_ - a) (E2).

On the other hand, following exponential growth equation fits values during recovery:

y = y_0_ + (a - y_0_) (1 - e ^-k·t^) (E3).

Derivative of E3 at time zero equals:

_dy/dt_| = k (a - y_0_) (E4).
